# Supplementary material for: Real-life evaluation of histologic scores for Ulcerative Colitis in remission
Source: PLoS One. 2021 Mar 8;16(3):e0248224. doi: 10.1371/journal.pone.0248224 (PMC7939352; doi:10.1371/journal.pone.0248224)

S1 Fig Difference between raters. Significant difference between raters were tested with wilcoxon rank sum test with Benjamini-Hochberg adjusted p-values

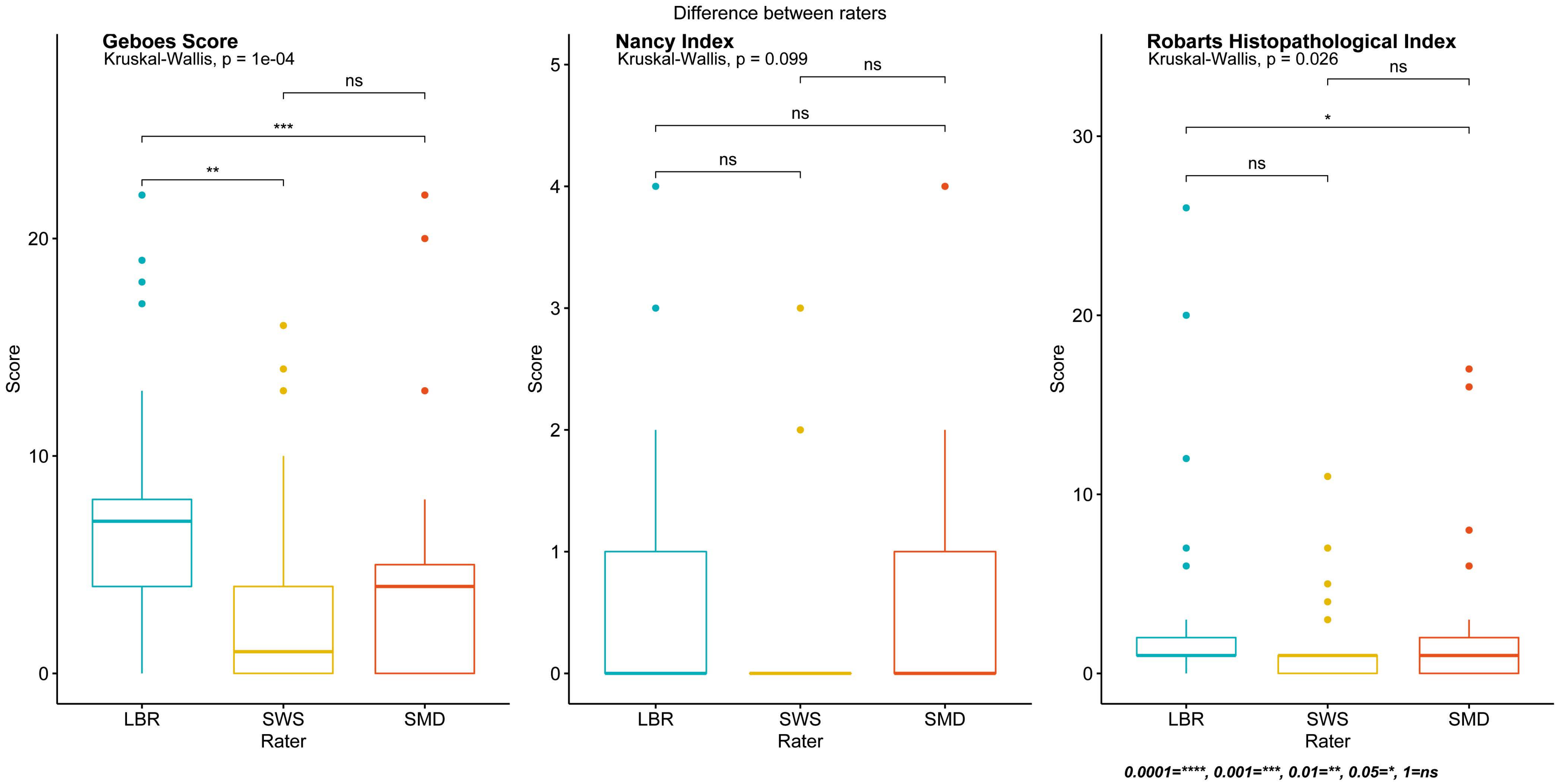

Supplement: S1 Fig — Significant difference between raters were tested with Wilcoxon rank sum test with Benjamini-Hochberg adjusted p-values. (PDF) [file pone.0248224.s001.pdf]
