# Supplementary material for: Real-life evaluation of histologic scores for Ulcerative Colitis in remission
Source: PLoS One. 2021 Mar 8;16(3):e0248224. doi: 10.1371/journal.pone.0248224 (PMC7939352; doi:10.1371/journal.pone.0248224)

S2 Fig Bar plot. Difference in patients classified as remission or active according to relaxed or strict definition of remission

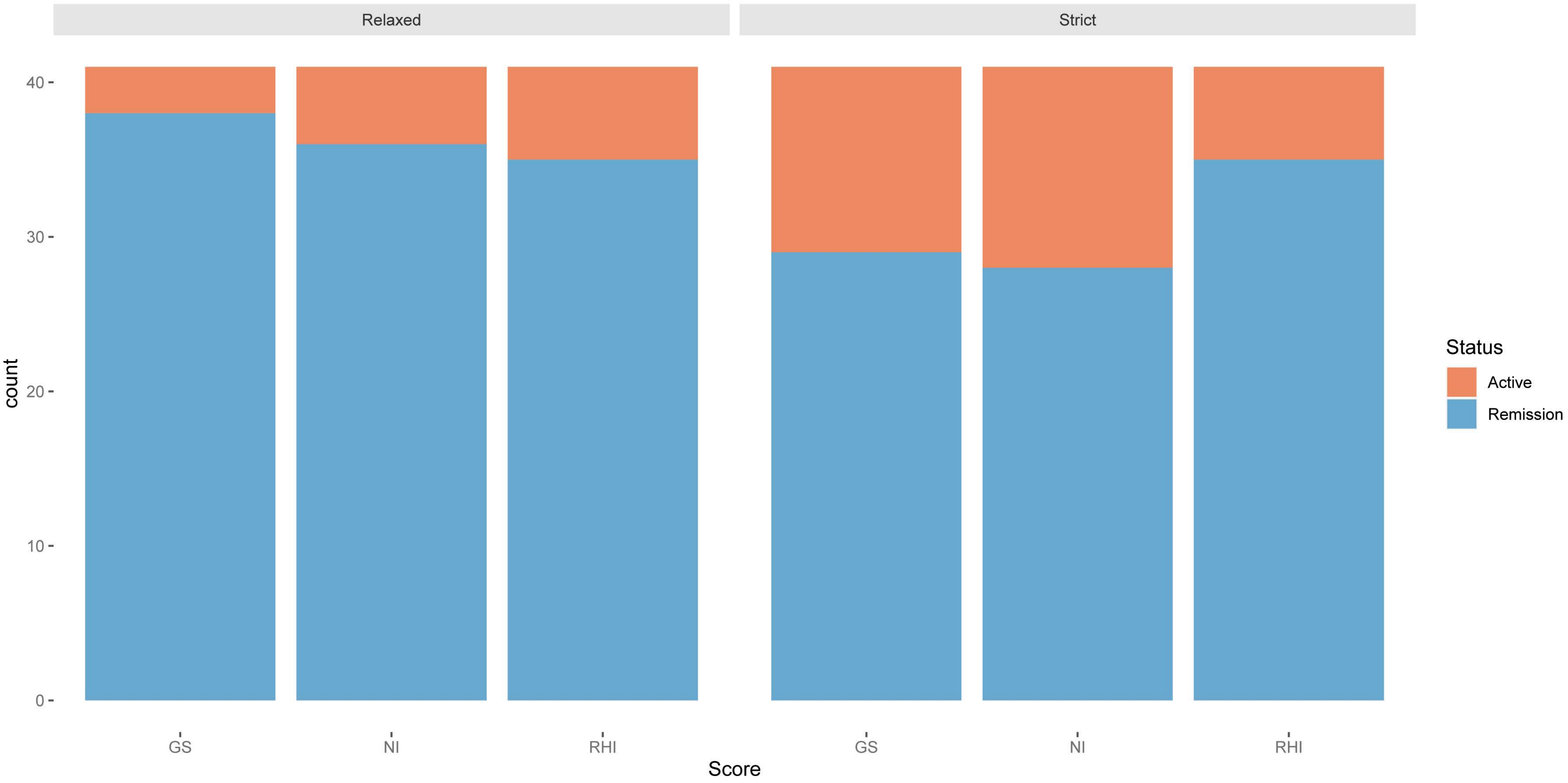

Supplement: S2 Fig — Difference in patients classified as remission or active according to relaxed or strict definition of remission. (PDF) [file pone.0248224.s002.pdf]
