# Supplementary figures and images for: Real-life evaluation of histologic scores for Ulcerative Colitis in remission
Source: PLoS One. 2021 Mar 8;16(3):e0248224. doi: 10.1371/journal.pone.0248224 (PMC7939352; doi:10.1371/journal.pone.0248224)

**S3 Fig Histologic score by biopsy location.** No difference was found between biopsy locations

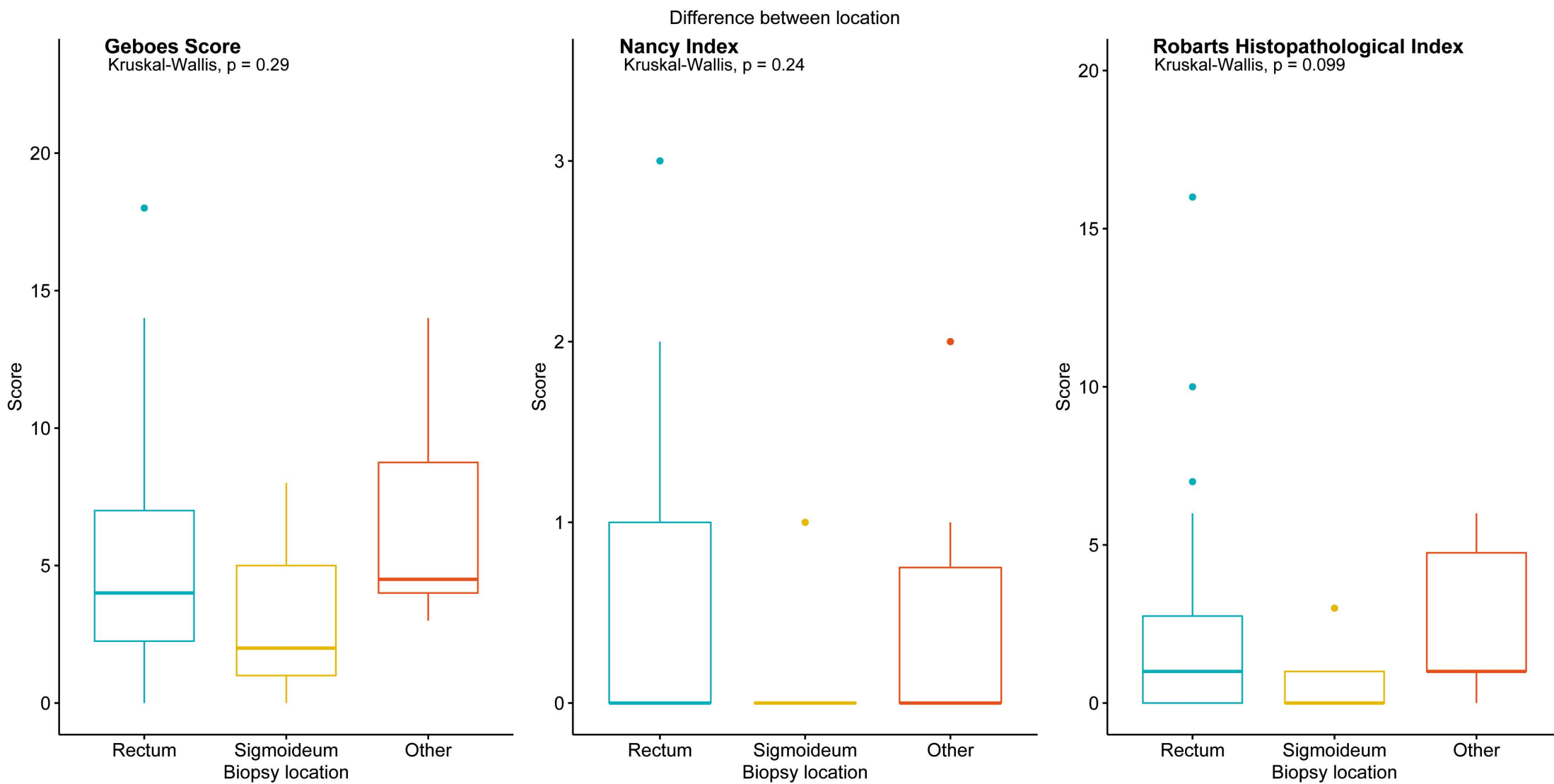

Supplement: S3 Fig — No difference was found between biopsy locations. (PDF) [file pone.0248224.s003.pdf]
