# Supplementary material for: Real-life evaluation of histologic scores for Ulcerative Colitis in remission
Source: PLoS One. 2021 Mar 8;16(3):e0248224. doi: 10.1371/journal.pone.0248224 (PMC7939352; doi:10.1371/journal.pone.0248224)
Supplement: S1 Table — (DOCX) [file pone.0248224.s004.docx]

**S1 Table: Descriptics for raters**

| Indices | Rater | Count | Mean | Sd | Median | IQR |
| --- | --- | --- | --- | --- | --- | --- |
| GS | LBR | 41 | 7.3 | 5.2 | 7 | 4 |
| GS | SMD | 41 | 4.4 | 5.2 | 4 | 5 |
| GS | SWS | 41 | 3.5 | 4.4 | 1 | 4 |
|  | **Rater** | **Count** | **Mean** | **Sd** | **Median** | **IQR** |
| RHI | LBR | 41 | 2.98 | 5.32 | 1 | 1 |
| RHI | SMD | 41 | 1.98 | 3.70 | 1 | 2 |
| RHI | SWS | 41 | 1.12 | 2.15 | 1 | 1 |
|  | **Rater** | **Count** | **Mean** | **Sd** | **Median** | **IQR** |
| NI | LBR | 41 | 0.63 | 1.13 | 0 | 1 |
| NI | SMD | 41 | 0.51 | 0.84 | 0 | 1 |
| NI | SWS | 41 | 0.29 | 0.81 | 0 | 0 |
