# Supplementary material for: Real-life evaluation of histologic scores for Ulcerative Colitis in remission
Source: PLoS One. 2021 Mar 8;16(3):e0248224. doi: 10.1371/journal.pone.0248224 (PMC7939352; doi:10.1371/journal.pone.0248224)
Supplement: S2 Table — (DOCX) [file pone.0248224.s005.docx]

|  | Statistics | P-value | Raters |
| --- | --- | --- | --- |
| GS | 18.421 | 1e-04 | LBR-SMD, LBR-SWS |
| RHI | 7.2799 | 0.02625 | LBR-SWS |
| NI | 4.6268 | 0.09893 |  |

**S2 Table Kruskal-Wallis rank sum test on raters, by indices**
