# Supplementary material for: Real-life evaluation of histologic scores for Ulcerative Colitis in remission
Source: PLoS One. 2021 Mar 8;16(3):e0248224. doi: 10.1371/journal.pone.0248224 (PMC7939352; doi:10.1371/journal.pone.0248224)
Supplement: S3 Table — The table shows that histological category is independent for whether the sample was collected from a MES/UCCS 0 or 1 patient. This was true for both Strict and Relaxed category. (DOCX) [file pone.0248224.s006.docx]

**S3 Table Fisher exact test between the UCCS and MES scores and histologic category.** The table shows that histological category is independent for whether the sample was collected from a MES/UCCS 0 or 1 patient**.** This was true for both Strict and Relaxed category

|  | Remission Cuff-off | Index | 95% conf interval of odd ratio | p-value |
| --- | --- | --- | --- | --- |
| UCCS | **Strict** | Nancy | 0.5-29.4 | 0,2 |
|  |  | Robarts | 0.2-27.9 | 0,3 |
|  |  | Geboes | 0.3-14.9 | 0,4 |
|  | **Relaxed** | Nancy | 0.0-16.0 | 1,0 |
|  |  | Robarts | 0.2-27.9 | 0,3 |
|  |  | Geboes | 0.0-57.6 | 0,4 |
| MES | **Strict** | Nancy | 0.1-72.8 | 1,0 |
|  |  | Robarts | 0.2-90.8 | 1,0 |
|  |  | Geboes | 0.0-40.6 | 1,0 |
|  | **Relaxed** | Nancy | 0.1-5.1 | 1,0 |
|  |  | Robarts | 0.0-6.4 | 1,0 |
|  |  | Geboes | 0.1-6.0 | 1,0 |
