# Supplementary material for: Real-life evaluation of histologic scores for Ulcerative Colitis in remission
Source: PLoS One. 2021 Mar 8;16(3):e0248224. doi: 10.1371/journal.pone.0248224 (PMC7939352; doi:10.1371/journal.pone.0248224)
Supplement: S4 Table — (DOCX) [file pone.0248224.s007.docx]

**S4 Table**. **Wilcoxon rank sum test on the effect of medication on histological scores.**

| Scores | Yes | No | statistic | p | p.signif |
| --- | --- | --- | --- | --- | --- |
| Mesalazine | | | | | |
| *Geboes* | 38 | 3 | 41 | 0.436 | ns |
| *Nancy* | 38 | 3 | 34.5 | 0.18 | ns |
| *Robarts* | 38 | 3 | 38 | 0.334 | ns |
| Steroids | | | | | |
| *Geboes* | 1 | 40 | 2.5 | 0,149 | ns |
| *Nancy* | 1 | 40 | 13,5 | 0,537 | ns |
| *Robarts* | 1 | 40 | 7 | 0,27 | ns |
| Azathiopurine/Mercaptopurine | | | | | |
| *Geboes* | 17 | 24 | 238 | 0,373 | ns |
| *Nancy* | 17 | 24 | 237,5 | 0,287 | ns |
| *Robarts* | 17 | 24 | 242 | 0,3 | ns |
| Methotrexate | | | | | |
| *Geboes* | 6 | 35 | 81,5 | 0,394 | ns |
| *Nancy* | 6 | 35 | 84 | 0,357 | ns |
| *Robarts* | 6 | 35 | 77,5 | 0,299 | ns |
| Biological agents | | | | | |
| *Geboes* | 27 | 14 | 186 | 0,945 | ns |
| *Nancy* | 27 | 14 | 211,5 | 0,461 | ns |
| *Robarts* | 27 | 14 | 196 | 0,852 | ns |
