# Supplementary material for: Real-life evaluation of histologic scores for Ulcerative Colitis in remission
Source: PLoS One. 2021 Mar 8;16(3):e0248224. doi: 10.1371/journal.pone.0248224 (PMC7939352; doi:10.1371/journal.pone.0248224)
Supplement: S1 Appendix — (DOCX) [file pone.0248224.s008.docx]

S1 Appendix

Scoring aid provided to the pathologists:

Geboes original paper(1)

Nancy Index original and a practical guide(2, 3)

Robarts original paper(4).

Electronic atlas and feature explanation submitted by Mosli et al. as supplementary to their Robarts original article. Data supplement 1 and 2. Available from: <https://gut.bmj.com/content/66/1/50#supplementary-materials> (Visited 27.01.21)

1. Geboes K, Riddell R, Ost A, Jensfelt B, Persson T, Lofberg R. A reproducible grading scale for histological assessment of inflammation in ulcerative colitis. Gut. 2000;47(3):404-9.

2. Marchal-Bressenot A, Salleron J, Boulagnon-Rombi C, Bastien C, Cahn V, Cadiot G, et al. Development and validation of the Nancy histological index for UC. Gut. 2017;66(1):43-9.

3. Marchal-Bressenot A, Scherl A, Salleron J, Peyrin-Biroulet L. A practical guide to assess the Nancy histological index for UC. Gut. 2016;65(11):1919-20.

4. Mosli MH, Feagan BG, Zou G, Sandborn WJ, D'Haens G, Khanna R, et al. Development and validation of a histological index for UC. Gut. 2017;66(1):50-8.

**Table A** Geboes Continuous vs Geboes original

| Geboes Continous | Geboes orginal | |
| --- | --- | --- |
|  | Grade 0 | Structural (architectural change) |
|  | Subgrades | |
|  | 0.0 | No abnormality |
| 1 | 0.1 | Mild abnormality |
| 2 | 0.2 | Mild or moderate diffuse or multifocal abnormalities |
| 3 | 0.3 | Severe diffuse or multifocal abnormalities |
|  | Grade 1 | Chronic inflammatory infiltrate |
|  | Subgrades | |
|  | 1.0 | No increase |
| 4 | 1.1 | Mild but unequivocal increase |
| 5 | 1.2 | Moderate increase |
| 6 | 1.3 | Marked increase |
|  | Grade 2 | Lamina propria neutrophils and eosinophils |
|  | 2A Eosinophils | |
|  | 2A. 0 | No increase |
| 7 | 2A.1 | Mild but unequivocal increase |
| 8 | 2A.2 | Moderate increase |
| 9 | 2A.3 | Marked increase |
|  | 2B Neutrophils | |
|  | 2B. 0 | None |
| 10 | 2B.1 | Mild but unequivocal increase |
| 11 | 2B.2 | Moderate increase |
| 12 | 2B.3 | Marked increase |
|  | Grade 3 | Neutrophils in epithelium |
|  | 3.0 | None |
| 13 | 3.1 | < 5% crypts involved |
| 14 | 3.2 | < 50% crypts involved |
| 15 | 3.3 | > 50% crypts involved |
|  | Grade 4 | Crypt destruction |
|  | 4.0 | None |
| 16 | 4.1 | Probable—local excess of neutrophils in part of crypt |
| 17 | 4.2 | Probable—marked attenuation |
| 18 | 4.3 | Unequivocal crypt destruction |
|  | Grade 5 | Erosion or ulceration |
|  | 5.0 | No erosion, ulceration, or granulation tissue |
| 19 | 5.1 | Recovering epithelium+adjacent inflammation |
| 20 | 5.2 | Probable erosion—focally stripped |
| 21 | 5.3 | Unequivocal erosion |
| 22 | 5.4 | Ulcer or granulation tissue |
